# Genome-Wide Analysis of Sorghum GT47 Family Reveals Functional Divergences of MUR3-Like Genes

**DOI:** 10.3389/fpls.2018.01773

**Published:** 2018-12-14

**Authors:** Hua Xu, Anming Ding, Sihui Chen, Prince Marowa, Dian Wang, Min Chen, Ruibo Hu, Yingzhen Kong, Malcolm O’Neill, Guohua Chai, Gongke Zhou

**Affiliations:** ^1^Key Laboratory of Biofuels, Qingdao Institute of Bioenergy and Bioprocess Technology, Chinese Academy of Sciences, Qingdao, China; ^2^Key Laboratory of Tobacco Gene Resources, Tobacco Research Institute of Chinese Academy of Agricultural Sciences, Qingdao, China; ^3^Shandong Provincial Key Laboratory of Plant Stress Research, College of Life Science, Shandong Normal University, Jinan, China; ^4^Complex Carbohydrate Research Center, University of Georgia, Athens, GA, United States

**Keywords:** *Sorghum*, GT47 family, genome-wide analysis, MUR3, functional divergences

## Abstract

Sorghum (*Sorghum bicolor*) is an important bioenergy crop. Its biomass mainly consists of the cellulosic and non-cellulosic polysaccharides, both which can be converted to biofuels. The biosynthesis of non-cellulosic polysaccharides involves several glycosyltransferases (GT) families including GT47. However, there was no systemic study on GT47 family in sorghum to date. Here, we identified 39 sorghum GT47 family members and showed the functional divergences of MURUS3 (MUR3) homologs. Sorghum GT47 proteins were phylogenetically clustered into four distinct subfamilies. Within each subfamily, gene structure was relatively conserved between the members. Ten gene pairs were identified from the 39 GT47 genes, of which two pairs might be originated from tandem duplication. 25.6% (10/39) of sorghum GT47 genes were homologous to Arabidopsis MUR3, a xyloglucan biosynthesis gene in primary cell walls. *SbGT47_2, SbGT47_7*, and *SbGT47_8*, three most homologous genes of *MUR3*, exhibited different tissue expression patterns and were selected for complementation into Arabidopsis *mur3-3*. Physiological and cell wall analyses showed that SbGT47_2 and SbGT47_7 may be two functional xyloglucan galactosyltransferases in sorghum. Further studies found that MUR3-like genes are widely present in the seed plants but not in the chlorophytic alga *Chlamydomonas reinhardtii*. Our results provide novel information for evolutionary analysis and functional dissection of sorghum GT47 family members.

## Introduction

Sorghum(*Sorghum bicolor*), a highly productive C_4_ photosynthetic grass, is the fifth most cultivated cereal crop globally due to its huge biomass yield, high nitrogen utilization efficiency and remarkable adaptability on marginal land (Taylor et al., 2010; Byrt et al., 2011). Sorghum germplasms have abundant genetic diversity and can be classified into grain, forage, energy, and sweet sorghum based on the growth characteristics and end-uses (Rooney et al., 2007). In comparison to lignocellulosic biomass crops such as switchgrass and Miscanthus, sorghum has a smaller genome (∼730 Mb) and more fermentable soluble sugars, making it an ideal model for functional analysis of C4 grasses (Paterson et al., 2009).

Sorghum biomass consists of 24–38% cellulose, 12–22% non-cellulosic polysaccharides, 17–22% lignin, and 1–22% starch (Corredor et al., 2009). Non-cellulosic polysaccharides generally interact with cellulose and lignin, contributing to the strength of plant cell walls (Scheller and Ulvskov, 2010). In dicots and non-commelinoid monocotyledons, the major non-cellulosic polysaccharides of primary walls are xyloglucans and a range of pectic polysaccharides, with lower levels of heteroxylans and heteromannans. In contrast, primary walls of grasses have much lower levels of xyloglucans and pectins, which are replaced by higher amounts of heteroxylans and, in some cases, with (1,3; 1,4)-β-glucans (Burton and Fincher, 2012). Charactirization of Arabidopsis mutants shows that a battery of glycosyltransferases (GTs) from family GT2, GT8, GT34, GT37, GT43, and GT47 are involved in the biosynthesis of non-cellulosic polysaccharides (Perrin et al., 1999; Faik et al., 2002; Madson et al., 2003; Bauer et al., 2006; Burton et al., 2006; Brown et al., 2007, 2009; Cocuron et al., 2007; Lee et al., 2007).

GT47 proteins participate in xylan and xyloglucan biosynthesis in plants. Arabidopsis IRREGULAR XYLEM10 (IRX10) and IRX10-L possess UDP-Xyl: β-(1→4)-xylosyl transferase activities *in vitro* and are essential for formation of xylan backbone in secondary cell walls (Brown et al., 2009; Wu et al., 2009; Jensen et al., 2014; Urbanowicz et al., 2014). FRAGILE FIBER8 (FRA8)/IRX7 is required for synthesis of the tetrasaccharide at the reducing end of glucronoxylan in secondary cell walls (Zhong et al., 2005). Other GT47 family members MURUS3 (MUR3), XYLOGLUCAN L-SIDE CHAIN GALACTOSYLTRANSFERASE2 (XLT2), XYLOGLUCAN D-SIDE-CHAIN TRANSFERASE (XDT), XYLOGLUCAN S-SIDECHAIN TRANSFERASE 1 (XST1) and XYLOGLUCAN-SPECIFIC GALACTURONOSYLTRANSFERASE 1 (XUT1) contribute to xyloglucan side chain synthesis (Madson et al., 2003; Jensen et al., 2012; Peña et al., 2012; Schultink et al., 2013; Zhu et al., 2018). Arabidopsis MUR3 adds a galactosyl unit solely to the third xylosyl residue from the non-reducing end in the XXXG xyloglucan (XyG) oligosaccharide core, producing XXLG (Reiter et al., 1997; Madson et al., 2003; Kong et al., 2015). Ectopic expression of *Eucalyptus grandis* *MUR3* in the Arabidopsis *mur3* restored the fucosylated side chains, indicating a functional conservation of XyG structure in dicots (Lopes et al., 2010). In rice, fucogalactosylated xyloglucan is retained only in specific tissues, such as the root epidermis, root tip-growing hairs and pollen tubes (Liu et al., 2015). Further, *OsMUR3* overexpression in *mur3.1xlt2* results in production of XXLG, XXFG and their O-acetylated forms, indicating that OsMUR3 is enzymatically equivalent to MUR3 (Liu et al., 2015).

Although the sorghum genome (v3.1.1) is recently released (McCormick et al., 2018), there are few functional studies in sorghum due to loss of high-efficient genetic transformation system. In this work, we reported the comprehensive genomic identification and phylogenetic analysis of 39 GT47 family members in sorghum, as well as their expression patterns in six different tissues. Three genes (*SbGT47_2, SbGT47_7*, and *SbGT47_8*) that are the most homologous to *MUR3* were selected for functional characterization. Genetic complementation and cell wall analysis showed that they differentially rescued phenotypic defects of *mur3-3*. At least two MUR3-like genes (*SbGT47_2* and *SbGT47_7*) are part of the synthetic machinery necessary to produce fucoglalactoxyloglucan in sorghum.

## Materials and Methods

### Identification of GT47 Proteins in Sorghum and Other Nine Species

To identify GT47 proteins in ten representative species namely green alga (*Chlamydomonas reinhardtii*), moss (*Physomitrella patens)*, sorghum (*S. bicolor*), stiff brome (*Brachypodium distachyon*), rice (*Oryza sativa*), switchgrass (*Panicum virgatum*), Arabidopsis (*Arabidopsis thaliana*), grape (*Vitis vinifera*), alfalfa (*Medicago truncatula)* and poplar (*Populus trichocarpa*), Pfam (PF03016) analysis was employed to search against their corresponding genome database^1^. All GT47 proteins were manually verified for the presence of exostosin domain using InterProScan program^2^. Online software FGENESH^3^ was used to correct the mis-annotated GT47 genes.

### Sequence Properties of Sorghum GT47 Proteins

The physicochemical parameters of sorghum GT47 proteins were calculated using DNAman software (Lynnon Biosoft, Canada). Transmembrane domain was predicted by TMHMM Server v. 2.0^4^. The exon/intron organization of genes was generated with Gene Structure Display Server (GSDS) ^5^. Tandem duplications was identified based on the criteria that two partners are separated by no more than five gene loci in a range of 100 kb distance (Chai et al., 2012).

### Phylogenetic Analysis

Full-length protein sequences were aligned by ClustalX (version 1.83). The phylogenetic tree was generated using the Neighbor-Joining (NJ) and Maximum Parsimony (MP) algorithms, respectively, in MEGA 4.0 (Tamura et al., 2007). Evaluation of the nodes significance was performed by bootstrap analysis with 1,000 replicates. Pairwise gap deletion mode was adopted to ensure that the divergent domains in the NJ tree.

### Quantitative Real Time PCR (qRT-PCR)

Young leaf, root, upper stem (internodes 6∼8), middle stem (internodes 3∼5) and basal stem (internodes 1∼2) were sampled from 3-month-old sorghum cultivar “Keller” grown in the greenhouse (16 h light/8 h dark, 25∼28°C). Three independent plants were selected for qRT-PCR analysis as biological repeats. Total RNA was isolated with the RNeasy mini kit (Qiagen, United States) and genomic DNA was removed with RQ1 RNase-Free DNase (Promega, United States). First-strand cDNA was synthesized using oligo (dT) primer and M-MLV RT (Promega, United States). Primers were designed by Beacon Designer v7.0 (Premier Biosoft International, United States) setting primer melting temperature as 58∼60°C, primer length as 20∼24 bp and amplicon length as 90∼150 bp. All primers (Supplementary Table S2) were rechecked with online software Primer-BLAST ^6^ in order to determine primer specificity. qRT-PCR was conducted on a LightCycler^®^480 detection system (Roche, Germany) with SYBR Premix Ex Taq (TaKaRa, Japan). *SbACTIN2* was used as an internal reference gene.

### Subcellular Localization of *SbGT47_2, SbGT47_7* and *SbGT47_8*

The *SbGT47_2, SbGT47_7*, and *SbGT47_8* coding regions were individually fused with GFP at their C-terminus and expressed under control of a 35S cauliflower mosaic virus (CaMV 35S) promoter in the modified pBI221-GFP vector (Chen et al., 2003). The cytoplasmic tail and transmembrane domain of GmMan1 was fused to mCherry at its C-terminus as Golgi marker (Nelson et al., 2007). Arabidopsis transient expression assay was performed following a method described previously (Chai et al., 2015). Protoplasts from 4-week-old rosette leaves were transformed with the PEG-mediated transformation method. The fluorescence was observed using FluoView FV1000 confocal microscope (Olympus, Japan) with an excitation of 488 nm and an emission of 510 nm for GFP, with an excitation of 580 nm and an emission of 610 nm for mCherry.

### Complementation of *mur3-3* by *SbGT47_2, SbGT47_7* or *SbGT47_8* Overexpression

The *SbGT47_2, SbGT47_7*, and *SbGT47_8* coding sequence were individually ligated into the pCAMBia1300-GFP vector (Kong et al., 2009) to generate the overexpression constructs. The resulting constructs were transformed into *Agrobacterium tumefaciens* strain (EHA105) by electroporation. After confirmation that the *A. tumefaciens* strain contained the proper gene, pCAMBia1300-gene-GFP was introduced into 5-week-old Arabidopsis *mur3-3* mutant by using *A. tumefaciens*–mediated floral dip method (Zhang et al., 2006). T_0_ transgenic plants were screened on 1/2 MS plates containing 20 mg/L hygromycin, and T_3_ homologous lines were used for subsequent analysis.

### Cell Wall Compositions

Rosette leaves of 4-week-old Arabidopsis plants and inflorescence stems of 7-week-old plants were sampled for quantification of cell wall compositions as previously described (Chai et al., 2015). To ensure the accuracy of the data, WT, *mur3-3* and three independent complemented lines for each *SbGT47* gene were selected. Alcohol insoluble residues (AIR) were prepared by gradient ethanol extraction of the sample powder for 30 min per gradient, and then soaked with 100% acetone for 2 h at 37°C. The starch in rosette leaves was degraded with α-amylase and amyloglucosidase (Sigma-Aldrich, United States).

To measure monosaccharide compositions, AIRs were hydrolyzed with 2 M trifluroacetic acid (TFA) at 120°C for 2 h (Balaghi et al., 2011; Yu et al., 2014). The hydrolysates were incubated with 1-phenyl-3-methyl-5-pyrazolone (PMP) for derivatization at 70°C for 30 min and extracted by chloroform for three times. The PMP derivatives were analyzed on a Waters high performance liquid chromatography (HPLC) system with a Thermo ODS-2 C18 column (4.6 × 250 mm) and a 2489 UV visible detector. To determine crystalline cellulose content, TFA-resistant materials were incubated with Updegraff reagent (acetic acid/nitric acid/water, 8:1:2 v/v) at 100°C for 30 min, and completely hydrolyzed with 72% H_2_SO_4_ (v/v) (Updegraff, 1969). Crystalline cellulose content was calculated by measurement of glucose level and a dehydration factor of 0.9 using a glucose assay kit (Cayman Chemical, United States) following our previous method (Chai et al., 2015). To detect lignin content, about 3 mg AIRs were solubilized by acetyl bromide solution, and 2 M sodium hydroxide and 0.5 M hydroxylamine hydrochloride were added to stop the reaction (Fukushima and Hatfield, 2001). Absorbance at 280 nm was recorded by an UV-visible spectrophotometer of VARIAN Cary 50 (VARIAN, United States). Percentage of acetyl bromide soluble lignin (% ABSL) was calculated by the formula (% ABSL = 0.236 × absorbance at 280 nm/weight of AIR).

### MALDI-TOF MS Analysis

XyG oligosaccharides (XyGOs) were obtained by treating suspensions of the AIR mentioned above and solutions of the 4 M KOH-soluble materials in 50 mm ammonium formate, pH 5, with 2 units of xyloglucan-specific endoglucanase (XEG) as described (Pauly et al., 1999). Ethanol was added to 70% (v/v) and the soluble fraction was concentrated to dryness. The residue was dissolved in water and repeatedly freeze-dried to ensure removal of ammonium formate. The residue was then dissolved in water (1 mL) and the XyGOs enriched using graphitized carbon (Packer et al., 1998).

MALDI-TOF MS was performed in the positive ion mode using a Bruker Microflex spectrometer and workstation (Bruker, Billerica, MA, United States). Solutions (5 μL) of XyGOs (∼1 mg/mL in water) were mixed with an equal volume of 10 mM NaCl. A portion of this mixture (1 μL) was then added to 0.1 M dihydroxybenzoic acid (1 μL, 10 mg/mL in aqueous 50% acetonitrile) on the MALDI target plate and concentrated to dryness under a flow of warm air. Spectra from at least 200 laser shots were summed up to generate each mass spectrum.

### Statistical Analysis

Data were statistically analyzed using the one-way analysis of variance (ANOVA). Asterisks denote significant differences between two groups of data (^∗^*P* < 0.05; ^∗∗^*P* < 0.01). Significant differences is represent by different letter in the analysis of cell wall compositions (*P* < 0.05).

## Results

### Identification and Phylogenetic Analyses of 468 GT47 Proteins From Ten Species

A total of 468 GT47 proteins were identified in an alga (38), moss (57), four members of the grass family (rice, 41; switchgrass, 75; sorghum, 39; and stiff brome, 34), and four dicots (Arabidopsis, 39; alfalfa, 48; poplar, 62; and grape, 35) (Supplementary Table S1). The 468 GT47 proteins can be divided into nine clades (GT47-A to F) in the phylogenetic analysis (Supplementary Figure S1). The numbers of GT47 proteins from sorghum and other three monocots were obviously larger than those from the dicots in GT47-A, -B, and -D2, but smaller in GT47-C1, -C2, and -C3, similar to the distribution of sorghum GT8, GT34, and GT37 family members (Rai et al., 2016). GT47-E covered almost all GT47 genes from the chlorophytic alga *C.* *reinhardtii*, but only one copy in each plant species except allotetraploid switchgrass, consistent with a recent report that the GT47 genes from *C.* *reinhardtii* are clustered into an ancient clade in phylogenetic analysis (Tan et al., 2018).

### Phylogenetic Analyses of the Sorghum GT47 Proteins

Thirty-nine sorghum GT47 proteins were named as from SbGT47_1 to SbGT47_39 following the previously available nomenclature (Chai et al., 2012). These proteins contained one exostosin motif and varied from 206 to 783 amino acids (aa) in length with an average of 485 aa. It’s noteworthy that 87.2% (34/39) of GT47 proteins had a transmembrane region. The details on other parameters of nucleic acid and protein sequences were provided in Table 1 and Supplementary Table S1.

**Table 1:** Sequence characteristics of 39 sorghum GT47 genes.

Gene symbol	Gene locus	CDS(bp)	Exons	Protein			Transmembrane
				Length(aa)	Mol. Wt (kD)	PI	
SbGT47_1	Sobic.001G228900.1	1380	2	459	51.10	8.59	1
SbGT47_2	Sobic.001G229000.1	1842	1	613	67.55	6.54	0
SbGT47_3	Sobic.001G229100.1	1473	1	490	54.41	9.14	0
SbGT47_4	Sobic.001G303300.1	1353	1	450	50.49	8.28	0
SbGT47_5	Sobic.001G387300.1	1389	2	462	52.03	7.85	1
SbGT47_6	Sobic.001G486900.1	2352	14	783	88.59	6.83	1
SbGT47_7	Sobic.001G506300.1	1851	1	616	69.75	5.81	1
SbGT47_8	Sobic.001G506500.1	1641	1	514	58.39	8.10	1
SbGT47_9	Sobic.001G506600.1	1563	2	520	58.52	8.47	1
SbGT47_10	Sobic.001G506700.1	1539	2	512	57.02	9.96	1
SbGT47_11	Sobic.001G506800.1	1590	2	529	58.95	9.78	0
SbGT47_12	Sobic.001G506900.1	1542	2	513	57.14	8.52	0
SbGT47_13	Sobic.001G538700.1	1290	3	429	48.55	9.85	1
SbGT47_14	Sobic.001G541600.1	621	1	206	22.70	6.50	0
SbGT47_15	Sobic.002G062100.1	1755	3	584	63.59	8.80	1
SbGT47_16	Sobic.002G342300.1	1371	3	500	55.79	9.70	1
SbGT47_17	Sobic.003G102700.1	1716	8	566	63.92	9.31	1
SbGT47_18	Sobic.003G234701.1	1515	4	307	35.59	10.3	0
SbGT47_19	Sobic.003G331000.1	1500	8	499	56.27	9.07	1
SbGT47_20	Sobic.003G360300.1	1281	8	426	48.56	9.91	1
SbGT47_21	Sobic.003G405600.1	1299	4	437	48.78	9.44	1
SbGT47_22	Sobic.003G410600.1	1248	2	420	47.28	6.57	1
SbGT47_23	Sobic.003G410700.1	1254	4	417	46.90	6.90	0
SbGT47_24	Sobic.003G410800.1	1263	4	420	47.15	6.90	1
SbGT47_25	Sobic.004G070100.1	1212	5	408	46.29	9.25	1
SbGT47_26	Sobic.004G159100.1	1299	4	437	48.93	8.15	1
SbGT47_27	Sobic.004G213500.1	1413	3	475	53.08	9.65	1
SbGT47_28	Sobic.006G059000.1	1293	4	435	48.64	7.15	1
SbGT47_29	Sobic.006G186100.1	1707	2	573	63.03	8.47	1
SbGT47_30	Sobic.006G186200.1	1440	1	484	53.04	8.93	1
SbGT47_31	Sobic.006G260900.1	1371	3	461	52.27	9.19	1
SbGT47_32	Sobic.007G139300.1	1572	2	528	59.29	10.3	1
SbGT47_33	Sobic.008G021000.1	1488	2	500	55.13	8.98	1
SbGT47_34	Sobic.008G077900.1	1572	2	528	58.96	6.03	1
SbGT47_35	Sobic.008G138100.1	1761	1	591	65.75	9.25	1
SbGT47_36	Sobic.009G162700.1	1269	2	427	47.54	6.83	1
SbGT47_37	Sobic.009G220100.1	1248	1	420	46.81	6.83	1
SbGT47_38	Sobic.009G220200.1	1251	4	421	47.51	6.94	0
SbGT47_39	Sobic.010G059400.1	1530	4	551	61.69	9.84	0

*Molecular Wt and PI of sorghum GT47 proteins have been calculated using DNAman software. Transmembrane domain is predicted by TMHMM Server v. 2.0*.

**FIGURE 1:**
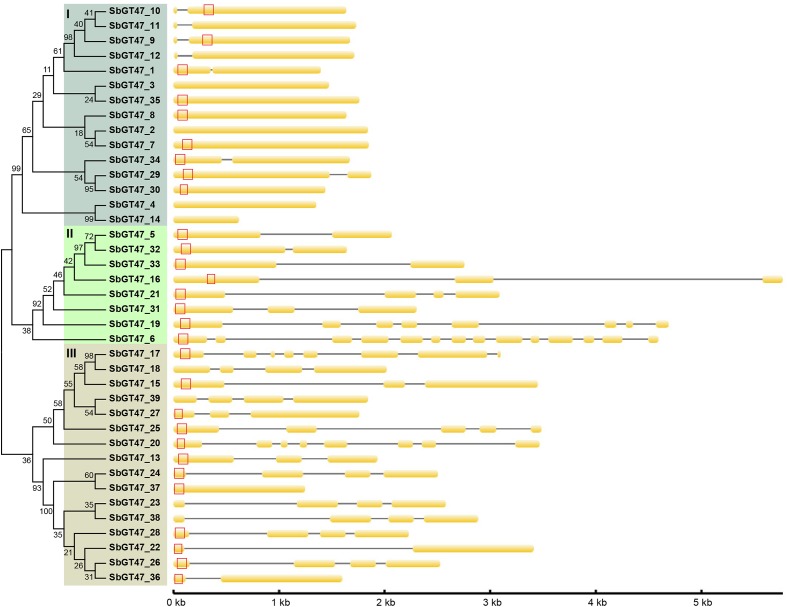
Phylogenetic relationship and gene structure of sorghum GT47 genes. Full-length proteins were aligned with Clustal X 1.83 and the phylogenetic tree was constructed using MEGA 4.0 by the method of Neighbor-Joining (NJ) with 1,000 bootstrap replicates. Orange box and black line show exon and intron, respectively. Transmembrane domain predicted by TMHMM Server v. 2.0 (http://www.cbs.dtu.dk/services/TMHMM/) is shown with red box. Gene size can be estimated by the scale (bottom).

To evaluate the evolutionary relationships between sorghum GT47 members, a phylogenetic analyze was performed based on full-length protein or exostosin motif sequences. Phylogenetic trees generated by the NJ and MP algorithms were largely comparable with only minor modifications at interior branches (Figure 1 and Supplementary Figure S1). The 39 GT47 proteins were generally divided into four subfamilies (I-IV). Within each subfamily, most closely related members shared similar exon/intron structures either in terms of intron numbers or exon length, which was roughly cconsistent with the classification defined in the phylogenetic tree. For instance, the GT47 genes in subfamily I contained zero or one intron, while those in subfamily IV possessed two to three introns with the exception of *SbGT47_36* and *SbGT47_37*. In addition, ten gene pairs were identified from the 39 GT47 genes based on the >50% bootstrap values in phylogenetic analysis (Figure 1).

### Chromosomal Location of the 39 GT47 Genes

**FIGURE 2:**
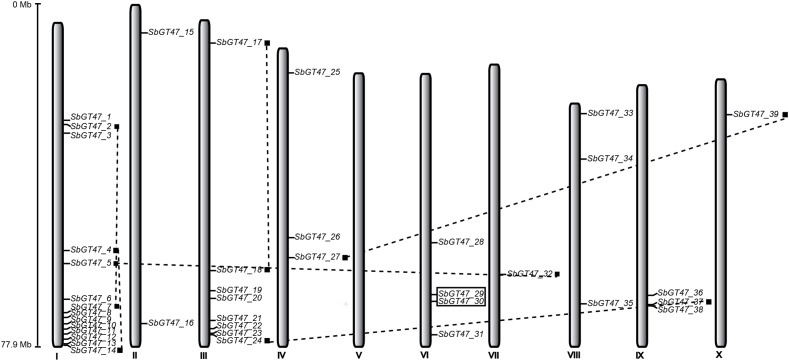
Chromosomal locations of sorghum GT47 genes. Eight of ten putative gene pairs are connected by dashed lines, other two resulted from tandem duplication are encompassed in black boxes. Scale represents the length of chromosome II.

The 39 sorghum GT47 genes were located on 10 linkage groups (LG) of sorghum chromosome (Figure 2). Their distributions among the chromosomes appeared to be uneven: LG II, IV, VI, VII, VIII, IX, and X harbor no more than four GT47 genes, while relatively high densities of GT47 genes (22/39, 56.4%) were discovered in some locations on LG I and III. Ten GT47 gene pairs were widely distributed on 7 chromosomes. Of them, two counterparts of two adjacent gene pairs (*SbGT47_10/SbGT47_11* and *SbGT47_29/SbGT47_30*) have high protein sequence similarities (>70%) calculated by the Smith-Waterman algorithm^7^ and were distributed within a distance less than 6.5 kb on a chromosome (Figure 2), indicating that they may originate from tandem duplication during the evolutionary process.

### Expression Patterns of Sorghum GT47 Genes in Various Tissues

**FIGURE 3:**
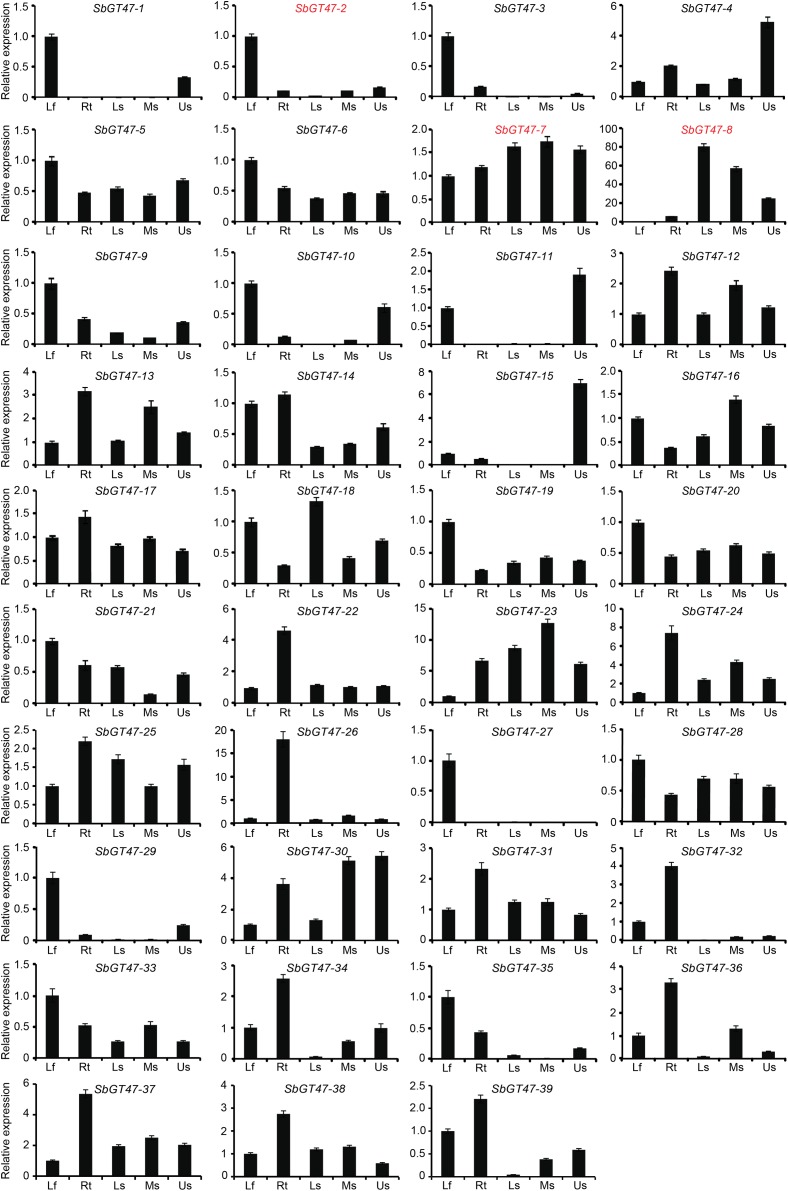
Tissue expression profiles of sorghum GT47 genes detected by qRT-PCR. SbGT47_2, SbGT47_7, and SbGT47_8 marked with red were selected for subsequent analysis. Lf, leaves; Rt, roots; BS, basal stems; MS, middle stems; US, upper stems. SbACTIN2 was used as an internal control. Bar represents standard deviations (SD) from three biological repeats.

To gain insight into the tissue-specific expression patterns of the 39 sorghum GT47 genes, qRT-PCR assays were performed in young leaves, roots, basal stems, middle stems, and upper stems of sorghum plants (Figure 3). 41% (16/39) of genes displayed tissue-specific expression patterns. Of them, eight genes (*SbGT47_1, SbGT47_2, SbGT47_3, SbGT47_9, SbGT47_10, SbGT47_27, SbGT47_29*, and *SbGT47_35*) were specifically expressed in young leaves, six genes (*SbGT47_22, SbGT47_24, SbGT47_26, SbGT47_32, SbGT47_34*, and *SbGT47_36*) in roots, and two genes (*SbGT47_8* and *SbGT47_15*) in stems. Interestingly, eight genes displayed distinct expression patterns across stem segments. *SbGT47_1, SbGT47_4, SbGT47_10, SbGT47_11, SbGT47_15*, and *SbGT47_29* were predominantly expressed in the upper stem, whereas transcripts of *SbGT47_8* and *SbGT47_18* were gradually increased from the upper to basal stem.

Tissue expression patterns of ten pairs of genes were obviously classified into two categories (Figure 3). The first category was composed of four gene pairs (*SbGT47_10/11, SbGT47_3/35, SbGT47_17/18*, and *SbGT47_24/18*), of which two gene duplicates exhibited similar expression patterns with respect to the tissues examined, implying the functional similarity between each other. In the second category, both duplicates of six gene pairs (*SbGT47_2/7, SbGT47_29/30, SbGT47_4/14, SbGT47_5/32, SbGT47_29/27*, and *SbGT47_23/38*) shared divergent expression patterns. For example, *SbGT47_2* was preferentially expressed in leaves, while *SbGT47_7* was constitutively expressed in all six tissues detected. This suggests that two counterparts in the six gene pairs might have undergone substantial divergence after duplication.

### Functional Analysis of Three Sorghum MUR3-Like Genes

**FIGURE 4:**
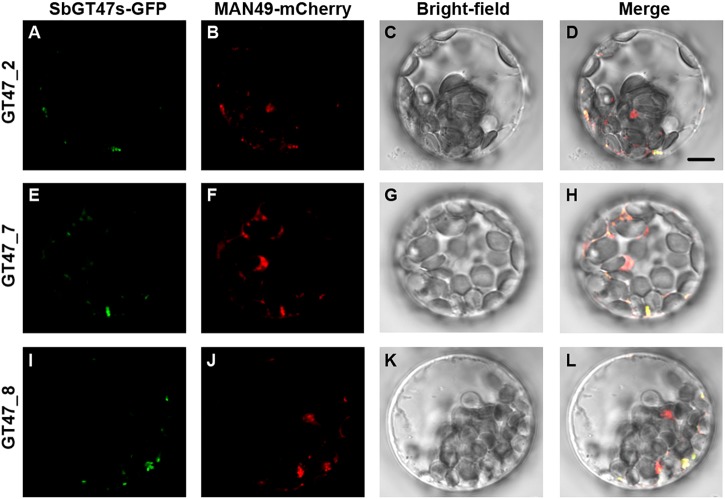
Subcellular localization of SbGT47_2, SbGT47_7 and SbGT47_8. SbGT47s-GFP and MAN49-mCherry (a Golgi marker) were transiently co-expressed in Arabidopsis leaf protoplasts. The localization of SbGT47_2 **(A)**, SbGT47_7 **(E)** and SbGT47_8 **(I)** were detected by green fluorescence, while Golgi was marked by MAN49 with red fluorescence **(B,F,J)**. Besides, bright-field images **(C,G,K)** and merged images **(D,H,L)** were also acquired for subcellular localization analysis. Bar = 10μm.

Arabidopsis MUR3 is involved in the modification side chain substitutions of xyloglucan, which has important roles in the formation of structural maintenance of primary cell walls (Madson et al., 2003; Kong et al., 2015). Here, we identified ten orthologs (SbGT47_1, _2, _3, _7, _8, _9, _10, _11, _12, and _35) of MUR3 in sorghum and found that they displayed distinct expression patterns across various tissue (Figure 3). To investigate putative divergences in their functions, SbGT47_2, SbGT47_7 and SbGT47_8, three most homologous genes of MUR3 (Supplementary Figure S1), were selected for subsequent analysis. Their subcellular localizations were first determined in Arabidopsis leave protoplasts. Similar to MUR3 (Chou et al., 2015), SbGT47_2-GFP, SbGT47_7-GFP or SbGT47_8-GFP was perfectly co-expressed with MAN49-mCherry (a Golgi marker) (Figure 4), indicating that SbGT47_2, SbGT47_7 and SbGT47_8 are located in Golgi apparatus.

**FIGURE 5:**
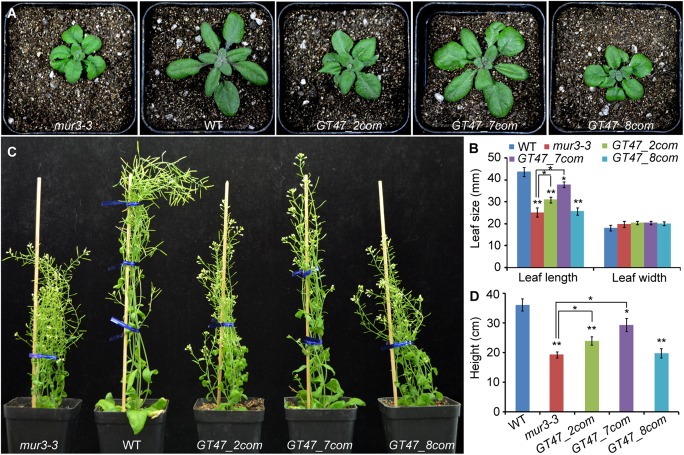
Phenotypes of four- **(A,B)** and 7-week-old **(C,D)** WT, mur3-3 and representative transgenic plants overexpressing SbGT47_2 (GT47_2com), SbGT47_7 (GT47_7com) or SbGT47_8 (GT47_8com) in mur3-3. For each genotype, at least 20 independent plants were used for measurement of leaf size and plant height. Asterisks represent the significant difference between two groups (^∗^P < 0.05; ^∗∗^P < 0.01).

To determine the biological functions of *SbGT47_2, SbGT47_7*, and *SbGT47_8*, their overexpression constructs were individually introduced into Arabidopsis *mur3-3* to generate transgenic plants called *GT47_2com, GT47_7com* and *GT47_8com*. At least 30 transgenic lines for each construct were obtained and the expression of these genes was confirmed by using RT-PCR (Supplementary Figure S2). T_3_ homozygous transgenic lines for each construct exhibited similar phenotypes. The cabbage-like and dwarf phenotypes of *mur3-3* were largely rescued by *SbGT47_7* overexpression, slightly rescued by *SbGT47_2* overexpression, but not affected by *SbGT47_8* overexpression, which were supported by the statistic data of leaf length and height in the transgenic plants (Figure 5). These results indicated functional divergences between sorghum SbGT47_2, SbGT47_7, and SbGT47_8. It is noteworthy that there was no observable difference for leaf width between wild-type, *mur3-3* and transgenic plants (Figure 5B).

To investigate whether overexpression of *SbGT47_2, SbGT47_7*, or *SbGT47_8* in *mur3-3* alters the composition of cell walls, monosaccharide composition analysis was performed in leaves and stems. The level of fucose, a major side-chain component of xyloglucans (XyG) solubilized from primary cell wall material, was dramatically reduced in AIR of the *mur3-3* leaves compared with the wild type (Table 2). Similarly, galactose content was also reduced in the *mur3-3* leaves. The relative abundances of all other detected monosaccharaides (xylose, arabinose, glucose, mannose, rhamnose and glucuronic acid) were not significantly different between two genotypes. These results were consistent with a previous report (Madson et al., 2003). In leaves, ectopic expression of *SbGT47_7* in *mur3-3* restored fucose and galactose contents to wild type-like level, whereas only fucose content was recovered by *SbGT47_2* overexpression. In contrast, the reduced fucose and galactose levels in *mur3-3* were not changed by *SbGT47_8* overexpression. These results strongly suggest that the three sorghum MUR3-like proteins may have different GT activities in leaves. Interestingly, mutation of *MUR3* led to significant alterations of xylose, arabinose, glucose, mannose and fucose contents in stems (Table 2). The contents of the five monosaccharaides in *mur3-3* were recovered by *SbGT47_2* and *SbGT47_7* overexpression to varying degrees, while only xylose was restored by *SbGT47_8* overexpression. WT and *mur3-3* showed similar galactose levels in 7-week-old inflorescence stems, possibly due to the dominance of other galactose containing polymers in secondary cell walls. Compared with the wild type, the levels of cellulose and lignin, other two major components of secondary cell walls, were markedly reduced in the *mur3-3* leaves or stems. Overexpression of *SbGT47_2, SbGT47_7*, or *SbGT47_8* in *mur3-3* resulted in an increase in cellulose and lignin contents to various extents.

**Table 2:** Cell wall compositions of wild-type, *mur3-3* and complemented transgenic plants.

									Galacturonic	Glucuronic
Tissue	Samples	Cellulose	Xylose	Arabinose	Glucose	Mannose	Galactose	Rhamnose	acid	acid	Fucose	Lignin
Leaf	WT	27.3±2.59^b^	13.8±1.34^a^	10.3±0.52^a^	26.5±1.08^a^	23.3±1.17^a^	31.2±1.62^a^	12.3±0.98^a^	45.0±2.03^a^	5.1±0.48^a^	4.1±0.36^a^	34.1±1.30^a^
	*mur3-3*	22.4±1.36^c^	14.0±3.51^a^	10.1±1.54^a^	29.0±2.04^a^	19.7±2.31^a^	25.5±1.82^b^	12.5±0.85^a^	43.9±4.97^a^	6.0±.059^a^	1.7±0.36^c^	29.2±2.09^b^
	SbGT47_2^com^	30.0±3.02^ab^	14.1±0.68^a^	10.7±0.21^a^	28.6±0.27^a^	20.4±2.95^a^	28.4±0.87^b^	12.5±0.38^a^	42.6±2.91^a^	5.4±0.79^a^	2.7±0.05^b^	32.0±2.95^a^
	SbGT47_7^com^	34.1±2.89^a^	11.7±1.32^a^	10.1±1.07^a^	27.7±1.93^a^	20.5±2.10^a^	29.8±1.41^a^	11.2±0.74^a^	43.6±3.52^a^	5.8±0.37^a^	3.7±0.20^a^	35.1±3.78^a^
	SbGT47_8^com^	27.0±0.78^b^	12.3±0.75^a^	11.4±0.46^a^	24.8±1.74^a^	23.3±2.34^a^	26.8±1.89^b^	12.5±0.73^a^	39.7±4.11^a^	5.9±0.60^a^	2.2±0.21^c^	34.8±3.07^a^
Stem	WT	484.5±28.4^ab^	208.2±4.59^a^	10.7±0.47^d^	13.8±1.08^c^	36.3±2.46^a^	19.7±0.70^a^	17.1±0.12^a^	45.3±3.97^a^	2.8±0.42^a^	1.5±0.13^a^	60.9±5.78^a^
	*mur3-3*	333.1±25.7^c^	159.7±8.62^c^	20.5±1.56^a^	21.2±2.65^ab^	28.5±1.05^b^	19.2±1.16^a^	15.8±2.10^a^	46.5±3.38^a^	2.2±0.55^a^	0.5±0.05^c^	48.8±2.92^c^
	SbGT47_2^com^	463.7±16.5^ab^	186.2±9.28^b^	18.4±0.36^b^	17.1±0.77^b^	29.1±1.92^b^	19.1±0.23^a^	16.1±0.73^a^	43.8±3.17^a^	2.0±0.32^a^	0.7±0.10^b^	51.3±3.39^bc^
	SbGT47_7^com^	498.3±11.6^a^	189.1±4.50^b^	13.5±0.93^c^	15.9±2.46^bc^	30.6±2.43^ab^	19.4±1.14^a^	17.5±1.54^a^	45.5±2.04^a^	3.1±0.85^a^	1.6±0.22^a^	52.0±1.48^bc^
	SbGT47_8^com^	435.2±19.3^b^	189.6±8.12^b^	20.88±0.54^a^	21.7±0.98^a^	29.1±2.48^b^	20.3±0.14^a^	17.4±1.64^a^	46.0±2.51^a^	2.6±0.29^a^	0.5±0.10^c^	55.5±2.67^b^

*Values are mean ± SE (*n* = 3) with the unit (mg/g). Means with the different letter are significantly different (One-way ANOVA, Duncan’s test, *p* < 0.05)*.

### MALDI-TOF MS Analysis of XyG Structure in Three MUR3-Like Complemented Plants

**FIGURE 6:**
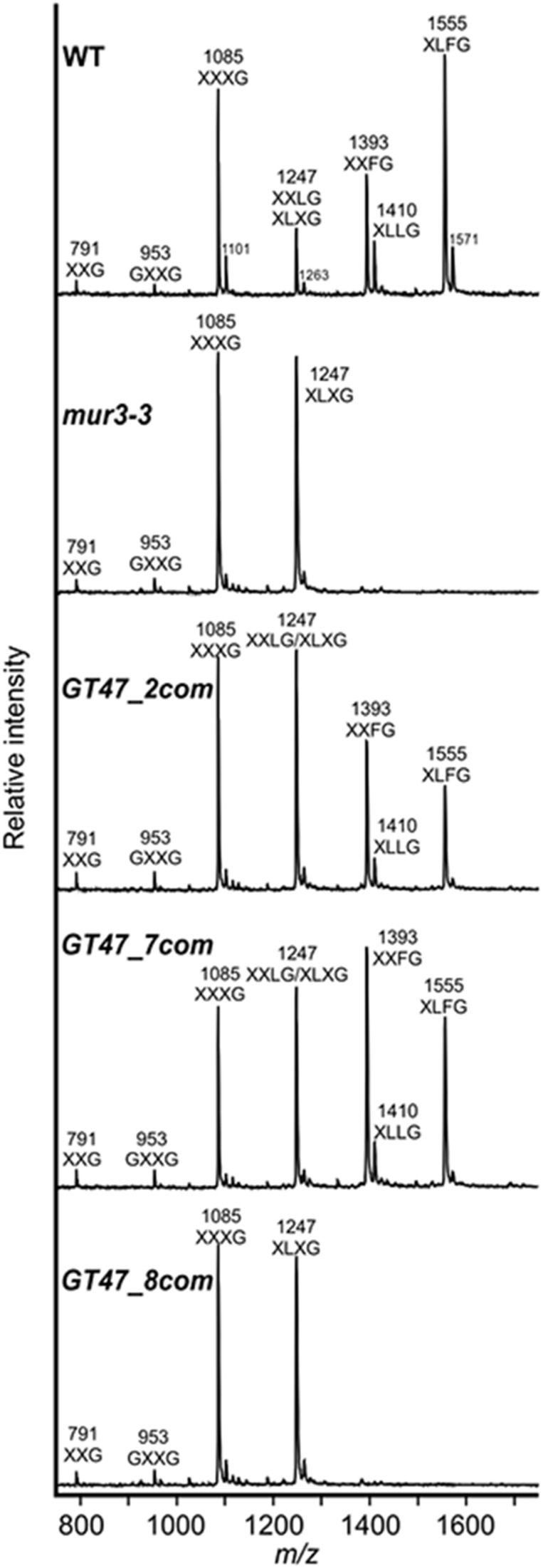
XyG MALDI-TOF mass spectra derived leaf cell wall material of 4-week-old WT, mur3-3, GT47_2com, GT47_7com, and GT47_8com. Ions representing known XyGOs are labeled using the one-letter code according to the nomenclature reported by Tuomivaara et al. (2015).

To determine whether SbGT47_2, SbGT47_7 or SbGT47_8, like MUR3, affects XyG structure, XyG oligosaccharides (XyGOs) derived from leaf AIRs of 4-week-old WT, *mur3-3* and complemented plants were analyzed by MALDITOF MS. Consistent with a previous report (Kong et al., 2015), all galactosylation (XXLG) and fucosylation (XXFG and XLFG) ions were lost in *mur3-3* (Figure 6). In the SbGT47_2 and SbGT47_7 complemented plants, the peaks corresponding to m/z 1,247 (XXLG/XLXG), 1,393 (XXFG), 1,410 (XLLG), and 1,555 (XLFG) were restored, confirming that SbGT47_2 and SbGT47_7 possess a xyloglucan galactosyltransferase activity. In contrast, the XyG structure was not restored in SbGT47_8 complemented plants, indicating that this complemented line cannot be considered to contain a functional MUR3.

## Discussion

In dicot plants, xyloglucan is one of major non-cellulosic polysaccharides in primary cell walls (Gibeaut et al., 2005). Arabidopsis MUR3 transforms the galactosyl residue from UDP-galactose to the third xylose (L side chain) of XXXG-type, forming XXLG subunits (Madson et al., 2003; Jensen et al., 2012). MUR3Gal is often substituted at O-2 with a α-L-Fuc*p* residue (F side chain: XXFG and XLFG) by GT37 family member FUCOSYLTRANSFERASE1 (FUT1) (Perrin et al., 1999). In this study, forty-six orthologs of MUR3 were identified in the seed plants selected, one in moss (Zhu et al., 2018), but none in the chlorophytic alga *C.* *reinhardtii* (Supplementary Figure S1). Mikkelsen et al. (2014) reported that the next level of xyloglucan side-chain biosynthesis genes are present in the charophytic algae *Penium margaritaceum*, which are the closest living relatives of land plants (Nishiyama et al., 2018). It is possible that glalactoxyloglucan is synthesized by enzymes with the MUR3 activity within charophyte algae, similar to the origination of plant 1,4-β-xylan (Jensen et al., 2018). At least seven orthologs of MUR3 were found in each of the four monocots, and drastically more than those (1–2 orthologs) in each of the four dicots, indicating that gene duplication may play a major role in the expansion of MUR3 in monocots. In addition to MUR3, XLT2 adds galactose to the second xylosyl residue of XXXG, producing XLXG, and XUT and XDT add galacturonic acid and arabinopyranose, respectively, to the O2-position of a xylosyl residue on xyloglucan (Jensen et al., 2012; Peña et al., 2012; Zhu et al., 2018). Here, 15 orthologs of XLT2 were found in all species except algae, 6 orthologs of XUT in dicots, one ortholog of XDT in moss but no ortholog of XST in all species detected (Supplementary Figure S1). These results suggest the specificity of side chain residue in xyloglucan structure among species.

Sorghum has ten orthologs of MUR3 and eight orthologs of IRX10/IRX10L, accounting for 46% of 39 GT47 genes (Supplementary Figure S1), suggesting the functional redundancy of these genes in xyloglucan and xylan biosynthesis in sorghum. 90% (9/10) of MUR3-like genes were arranged in clusters on LG I, and displayed various tissue expression patterns (Figures 2, 3). Physiological and cell wall analysis of transgenic plants expressing *SbGT47_2, SbGT47_7*, or *SbGT47_8*, three most homologous genes of *MUR3*, in *mur3-3* confirmed that SbGT47_2 and SbGT47_7 represent functional equivalent to MUR3 (Figures 5, 6 and Table 2). *SbGT47_7* showed stronger capability complement to *mur3-3* than *SbGT47_2*, correlating with its higher similarity to *MUR3* in both nucleotide sequence and tissue expression pattern (Schmid et al., 2005). Therefore, SbGT47_7 can be considered a fully functional xyloglucan MUR3 ortholog. These results suggest that the biosynthesis of xyloglucan structure in primary cell walls of sorghum may require MUR3-like genes, at least *SbGT47_2* and *SbGT47_7*. Also, sorghum, like rice (Liu et al., 2015), may have the ability to synthesize fucogalactoXyG *in vivo*.

Overexpressing tomato *SlMUR3* or rice *OsMUR3* in the Arabidopsis *xlt2mur3.1* mutant leads to a rescue of the dwarfed growth phenotype of plants (Schultink et al., 2013; Liu et al., 2015). Here, genetic and biochemical results showed that ectopic expression of sorghum *SbGT47_7* in *mur3-3* fully complemented the cabbage-like phenotype and defect in XyG structure, and the levels of cellulose, xylose and lignin were also restored in the stems of complemented lines (Table 2). It is generally believed that altered abundance of secondary wall polymers in *mur3* is a consequence of the stunted growth but not a direct effect of gene dysfunction (Liu et al., 2015). Thus, SbGT47_7 may indirectly affect the accumulation of cellulose, xylan and lignin in secondary cell walls of sorghum stems.

## Author Contributions

HX and GC designed the experiments, interpreted the results and wrote the paper. SC, DW, and RH participated in the data mining. AD, PM, and MC helped in sorghum materials collection and qRT-PCR detection. MO’N performed the MALDI-TOF MS analysis. GZ and YK analyzed the cell wall compositions and critically revised the manuscript. All authors approved the final manuscript.

## Conflict of Interest Statement

The authors declare that the research was conducted in the absence of any commercial or financial relationships that could be construed as a potential conflict of interest.

## Footnotes

1 https://phytozome.jgi.doe.gov/pz/portal.html

2 http://www.ebi.ac.uk/Tools/InterProScan

3 http://linux1.softberry.com/berry.phtml

4 http://www.cbs.dtu.dk/services/TMHMM/

5 http://gsds.cbi.pku.edu.cn/

6 www.ncbi.nlm.nih.gov/tools/primer-blast/

7 http://www.ebi.ac.uk/Tools/psa/

## Supplementary Material

FIGURE S1 Phylogeny of GT47 homologous proteins from ten representative species.

FIGURE S2 Identification of *SbGT47_2, SbGT47_7* and *SbGT47_8* complemented transgenic plants.

TABLE S1 Protein sequences of GT47 family members from ten species (doi: 10.6084/m9.figshare.6958895).

TABLE S2 Primers in this study.
