# Supplementary material for: Genome-Wide Analysis of Sorghum GT47 Family Reveals Functional Divergences of MUR3-Like Genes
Source: Front Plant Sci. 2018 Dec 14;9:1773. doi: 10.3389/fpls.2018.01773 (PMC6302003; doi:10.3389/fpls.2018.01773)
Supplement: FIGURE S1 — Phylogeny of GT47 homologous proteins from ten representative species. [file Table_1.DOCX]

**
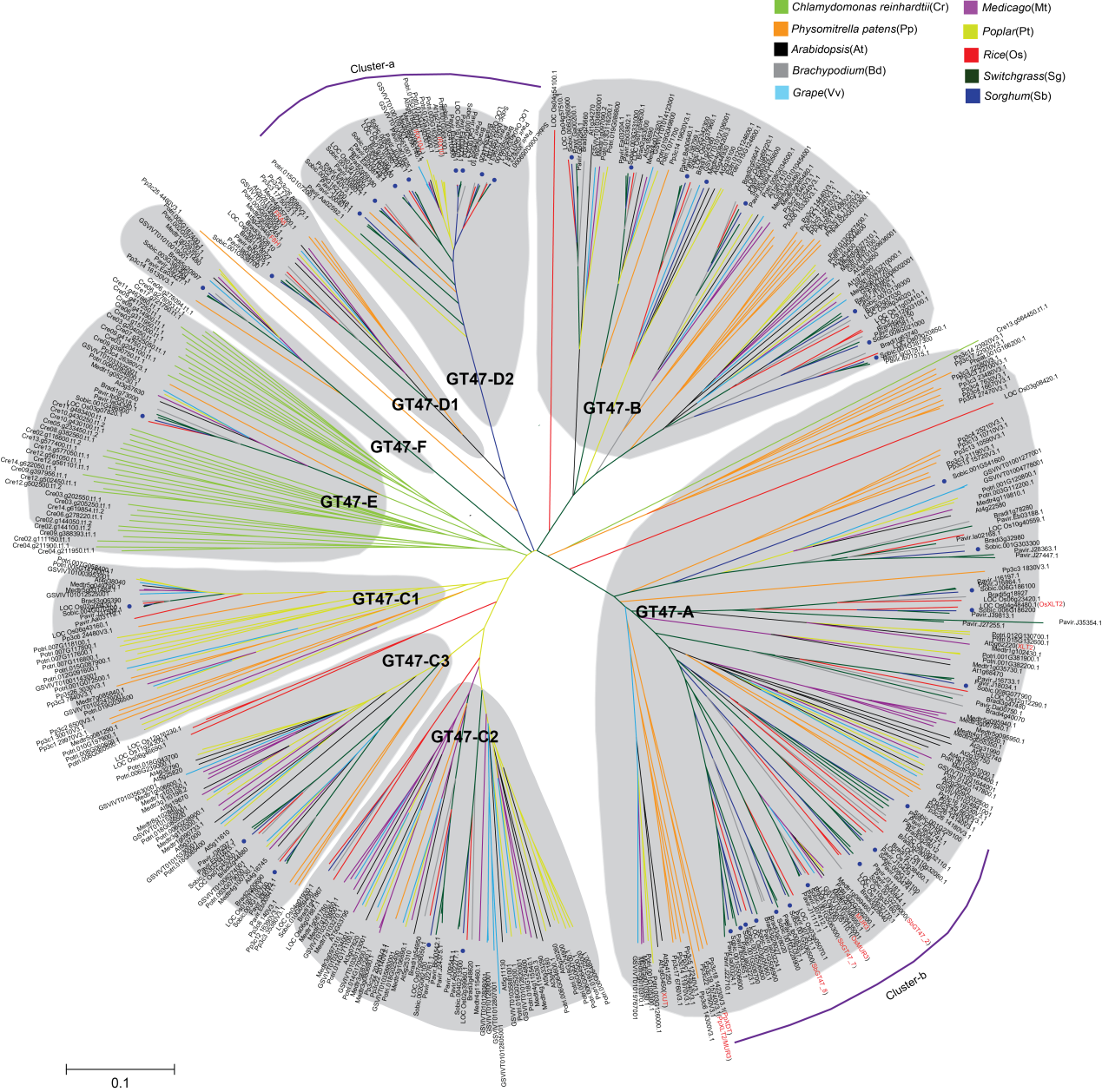
FIGURE S1** Phylogeny of 468 GT47 homologous proteins from ten representative species. Exostosin domain sequences were aligned by using online software muscle (<https://www.ebi.ac.uk>/Tools/msa/muscle/). Phylogenetic tree was constructed using MEGA 4.0 by the Neighbor-Joining (NJ) method with 1,000 bootstrap replicates. Seven known Arabidopsis hemicellulose-related proteins (MUR3, XLT2, XUT, XDT, IRX10, IRX10L, FRA8 and F8H) and three sorghum MUR3-like proteins (SbGT47_2, SbGT47_7 and SbGT47_8) are marked with red. Cluster-a, -b contain most of sorghum GT47 proteins. Bar represents evolutionary distance of 0.1 amino acid substitutions per site.


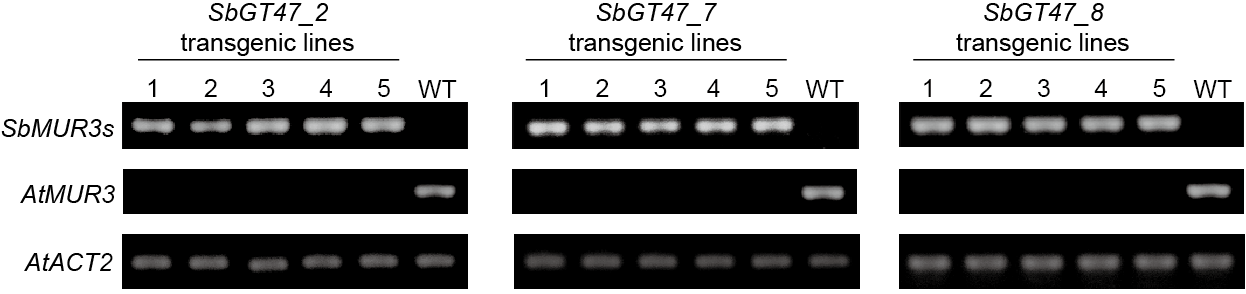


**FIGURE S2** Identification of representative *SbGT47_2*, *SbGT47_7* and *SbGT47_8* complemented transgenic lines. Different primers were designed to detect *MUR3* and *SbMUR3s* expression in wild-type and complemented plants. *AtACT2* (AT3G18780) was used as an internal control.
